# Clinical characteristics and mortality risk prediction model in children with acute myocarditis

**DOI:** 10.1007/s12519-022-00637-y

**Published:** 2022-11-15

**Authors:** Shi-Xin Zhuang, Peng Shi, Han Gao, Quan-Nan Zhuang, Guo-Ying Huang

**Affiliations:** 1 Children’s Hospital of Fudan University, 399 Wan Yuan Road, Shanghai, 201102 China; 2 Pediatric Clinical Research Unit, Department of Research Management, Children’s Hospital of Fudan University, Shanghai, China

**Keywords:** Acute myocarditis, Bayesian model averaging, Fulminant myocarditis, Hosmer–Lemeshow test, Mortality risk prediction model, Pediatrics

## Abstract

**Background:**

Acute myocarditis (AMC) can cause poor outcomes or even death in children. We aimed to identify AMC risk factors and create a mortality prediction model for AMC in children at hospital admission.

**Methods:**

This was a single-center retrospective cohort study of AMC children hospitalized between January 2016 and January 2020. The demographics, clinical examinations, types of AMC, and laboratory results were collected at hospital admission. In-hospital survival or death was documented. Clinical characteristics associated with death were evaluated.

**Results:**

Among 67 children, 51 survived, and 16 died. The most common symptom was digestive disorder (67.2%). Based on the Bayesian model averaging and Hosmer–Lemeshow test, we created a final best mortality prediction model (acute myocarditis death risk score, AMCDRS) that included ten variables (male sex, fever, congestive heart failure, left-ventricular ejection fraction < 50%, pulmonary edema, ventricular tachycardia, lactic acid value > 4, fulminant myocarditis, abnormal creatine kinase-MB, and hypotension). Despite differences in the characteristics of the validation cohort, the model discrimination was only marginally lower, with an AUC of 0.781 (95% confidence interval = 0.675–0.852) compared with the derivation cohort. Model calibration likewise indicated acceptable fit (Hosmer‒Lemeshow goodness-of-fit, *P*¼ = 0.10).

**Conclusions:**

Multiple factors were associated with increased mortality in children with AMC. The prediction model AMCDRS might be used at hospital admission to accurately identify AMC in children who are at an increased risk of death.

## Introduction

Acute myocarditis (AMC) is an inflammatory disease caused by various infections, with viruses being the most common pathogenic factor [1, 2]. The underlying pathophysiology of AMC might be due to direct damage from viral infection, postinfection inflammatory responses, or the combination of both. According to two national surveys in Japan, the annual incidence rates of myocarditis were 2.6 cases per 1,000,000 person-years and 3 cases per 1,000,000 person-years in 1997−2002 and 2006−2011, respectively [3, 4]. Some autopsy results showed a 0.1%−5.6% prevalence of AMC in both adults and children [5–7]. Myocarditis was also found at autopsy in 12% of teenagers who suffered sudden death [8]. Although the incidence rate varies among different populations, AMC can result in serious adverse consequences in affected children [2]. In a Japanese study, the mortality rate of children with AMC reached 14% [9].

The symptoms and signs of children with AMC are atypical and diverse, which leads to great challenges for timely diagnosis [10]. Most children with AMC initially present with upper respiratory tract or digestive tract infections, which can rapidly develop into heart failure, serious arrhythmia, and even sudden cardiac death [11]. Physical examinations are commonly nonspecific, although some children could have signs of heart failure. The electrocardiogram (ECG) might look similar to that of myocardial infarction, with increased myocardial enzymes. The most serious type of AMC is fulminant myocarditis, which usually presents as acute severe cardiac insufficiency, enlarged ventricles, pulmonary edema, and pericardial effusion [12]. Without prompt diagnosis and management, children with fulminant myocarditis could have high mortality. Thus, early recognition of AMC in children who are at a high risk of mortality is critical.

At present, there are limited studies showing the relationship between early presentation and mortality in children with AMC. Thus, we aimed to study the clinical presentation of AMC in children at the time of hospital admission. We explored the relationship between clinical presentation and death in children with AMC to identify variables that could predict their in-hospital deaths.

## Methods

### Study design and participants

This was a retrospective cohort study of children with AMC who were hospitalized at the Children’s Hospital of Fudan University, Shanghai, China, from January 2016 to January 2020. The study was approved by the Ethics Committee of the Children’s Hospital of Fudan University. Medical records were reviewed to select the study participants who met the selection criteria. The inclusion criteria were as follows: (1) age ≤ 18 years; and (2) a diagnosis of AMC, which was defined based on recent symptoms (< 10 days), clinical presentation, cardiac insufficiency, cardiogenic shock or cardio cerebral syndrome, cardiac enlargements, elevated troponin level, and ECG [13]. The exclusion criteria were as follows: (1) history of congenital heart disease, primary cardiomyopathy, rheumatic heart disease, other cardiac dysfunctions from metabolic disease, poisoning, hyperthyroidism, connective tissue disease, and other organ dysfunctions; (2) AMC as the secondary diagnosis; and (3) incomplete medical record.

### Data collection

Medical records were reviewed to collect information, including demographics, clinical presentations, laboratory test results, etiological examinations, ECG, and imaging findings, at the time of hospital admission. Enrolled children were assigned to either the survival or death groups based on their survival status at hospital discharge. Clinical data included gender, age, weight, medical history, symptoms, signs, length of hospital stay, and hospitalization expenses. Laboratory examinations included pathogen, white blood cell count, serum lactate level, and myocardial injury markers, such as cardiac troponin-I, creatine kinase-MB (CK-MB), and N-terminal pro-brain natriuretic peptide. Image studies included ECG and X-ray.

Fulminant myocarditis was defined as severe cardiac dysfunction or arrhythmia, cardiogenic shock requiring vasopressors with or without mechanical circulation support, or sudden cardiac death occurring within two weeks of symptom onset. Cardiogenic shock was defined as persistent hypotension (systolic blood pressure < 2 standard deviations for age) for at least one hour with no response to fluids or requiring a vasopressor to maintain blood pressure. Pulmonary edema was defined when the chest X-ray reported bilateral lung infiltration, with no clinical signs of infection. Ventricular tachycardia was defined as an ECG showing a ventricular rhythm of more than 110−120 beats per minute. Congestive heart failure was diagnosed based on clinical signs and symptoms, as well as chest X-ray, ECG, and laboratory results [13].

### Statistical analysis

All data were analyzed using the statistical software R language, version 3.5.3 (R Core Team, Vienna, Austria). Continuous data with a normal distribution are presented as the mean ± standard deviation. Categorical data are presented as numbers with percentages. Student’s *t* test was used to assess continuous data with normal distributions. Continuous data with a skewed distribution are presented as the median with interquartile range (IQR), which was analyzed using the Mann‒Whitney *U* test of two independent samples. Chi-square (*χ*^2^) or Fisher’s exact tests (with Bonferroni correction) were used to evaluate categorical data. After bivariate analysis, the variables with statistical significance (*P* < 0.05) were screened. A *P* value < 0.05 was considered statistically significant.

A multivariable model predicting survival was developed using Bayesian model averaging after multiple imputation [14]. Bayesian model averaging was performed on each of the imputed datasets, and the models were combined to form a final prediction model in which coefficients were averaged across the 20 models (Table 1). Model accuracy was assessed as the area under the curve (AUC). Bootstrapping was used to construct 95% confidence intervals (CIs) for the odds ratios. A score to predict the probability of survival was then constructed to allow individual predictions without the need for model refitting. A Hosmer–Lemeshow test was conducted for the final model to assess goodness of fit [2]. Predicted values of death were approximated into six equal parts and compared with observed values, along with a goodness-of-fit line. A *P* value > 0.05 suggests acceptable model fit. Finally, we used data from 22 children previously randomly selected in the database as an external validation database for the final model.

**Table 1:** Full multivariable prediction model

Variables	OR	SD	95% CI of OR
Gender (male)	1.12	0.08	0.96–1.28
Fever	1.24	0.12	1.00–1.48
Congestive heart failure	2.06	0.09	1.88–2.24
Left-ventricular ejection fraction < 50	2.33	0.11	2.11–2.55
Pulmonary edema	1.35	0.21	0.94–1.76
Ventricular tachycardia	1.46	0.32	0.83–2.09
Lactic acid value > 4	1.48	0.23	1.03–1.93
Fulminant myocarditis	3.15	0.43	2.31–3.99
CK-MB abnormal	1.25	0.12	1.01–1.49
Hypotension	1.55	0.15	1.26–1.84

*OR* odds ratio, *SD* standard deviation, *CI* confidence interval, *CK-MB* creatine kinase-MB

## Results

### Clinical symptoms and signs

There were 73 children diagnosed with AMC during the study period. Six children were excluded due to congenital heart diseases. In total, 67 children were included in our retrospective cohort study, with 51 children in the survival group and 16 children in the death group. The hospital stay in the survival group (median = 17.0, IQR = 9.0−24.0 days) was significantly longer than that in the death group (median = 2.0, IQR = 1.0−19.0 days) (*P* = 0.018). Otherwise, there were no statistically significant differences in age, gender, weight, or medical expenses between the two groups (Table 2).

**Table 2:** Baseline demographics and hospital admission information

Characteristics	Survival group (*n* = 51)	Death group (*n* = 16)	*t*/*χ*^2^	*P*
Age (y)	7.0 (5.0–10.0)	7.0 (2.0–11.0)	− 0.278	0.782
Weight (kg)	25.0 (17.0–36.0)	18.0 (10.0–35.0)	− 0.879	0.389
Sex, *n*				
Boy	28	10	0.286	0.593
Girl	23	6		
Hospitalized length (d)	17.0 (9.0–24.0)	2.0 (1.0–19.0)	− 2.537	0.018^*^
Medical expenses (RMB)	34,802.0 (9845.0–74,981.5)	25,692.0 (6500.5–104,816.5)	0.223	0.825

Values are presented as median (interquartile range). ^*^Statistically significant difference between two groups

As shown in Table 3, the most common clinical symptom was digestive system disorder (45 children, 67.2%), which was followed by circulatory system symptoms (29 children, 43.3%) and respiratory symptoms (17 children, 25.4%). There were no significant differences in the number of children with digestive, respiratory, and circulatory symptoms between the survival group and death group. There were significant differences in cardiogenic shock (*P* = 0.003) and hypotension (*P* = 0.007) between the survival group and death group. A total of 38 (56.7%) children had fulminant myocarditis, with 23 (45.1%) and 15 (93.8%) children in the survival and death groups, respectively (*P* = 0.001).

**Table 3:** Clinical presentations and examination results

Characteristics	Survival group	Death group	*t*/χ^2^/Fisher	*P*
Clinical signs and symptoms, *n* (%)	*n* = 51	*n* = 16		
Fever	32 (62.7)	5 (31.3)	4.886	0.027^*^
Digestive symptoms	35 (68.6)	10 (62.5)	0.207	0.649
Respiratory symptoms	14 (27.5)	3 (18.8)	0.487	0.485
Circulatory symptoms	23 (45.1)	6 (37.5)	0.286	0.593
Cardiogenic shock	20 (39.2)	13 (81.3)	8.610	0.003^*^
Adams-stokes	6 (11.8)	3 (18.8)	0.511	0.475
Respiratory distress	11 (21.6)	6 (37.5)	1.632	0.201
Cyanosis	3 (5.9)	3 (18.8)	2.473	0.116
Pale complexion	20 (39.2)	10 (62.5)	2.670	0.102
Hepatomegaly	4 (7.8)	3 (18.8)	1.549	0.213
Hypotension	9 (17.6)	9 (56.2)	9.237	0.002^*^
Fulminant myocarditis	23 (45.1)	15 (93.8)	11.743	0.001^*^
Pathogen examination, *n* (%)	*n* = 51	*n* = 15	1.049	0.306
Positive	22 (44.9)	9 (60.0)		
Negative	27 (55.1)	6 (40.0)		
Electrocardiographic, *n* (%)	*n* = 51	*n* = 14		
Abnormal ECG	51 (100.0)	13 (92.9)	3.700	0.054
Sinus tachycardia	15 (29.4)	3 (21.4)	0.350	0.554
Ventricular tachycardia	3 (5.9)	9 (64.3)	24.890	< 0.001^*^
Ventricular premature beat	7 (13.7)	3 (21.4)	0.501	0.479
Atrial premature beat	4 (7.8)	2 (14.3)	0.544	0.461
ST-T segment changes	26 (51.0)	3 (21.4)	3.882	0.049^*^
Degree I atrioventricular block	8 (15.7)	1 (7.1)	0.672	0.412
Degree II type 1 atrioventricular block	1 (2.0)	0 (0.0)	0.279	0.597
Degree II type 2 atrioventricular block	0 (0.0)	0 (0.0)	/	/
Degree III atrioventricular block	5 (9.8)	3 (21.4)	1.375	0.241
QT interval prolongation	2 (3.9)	0 (0.0)	0.566	0.452
Right bundle branch block	3 (5.9)	1 (7.1)	0.030	0.862
Left anterior branch block	4 (7.8)	0 (0.0)	1.170	0.279
Incomplete right bundle branch block	3 (5.9)	0 (0.0)	0.863	0.353
Intraventricular block	1 (2.0)	1 (7.1)	0.989	0.320
Atrial tachycardia	0 (0.0)	1 (7.1)	3.700	0.054
Atrial fibrillation	1 (2.0)	0 (0.0)	0.279	0.597
Junctional escape	1 (2.0)	0 (0.0)	0.279	0.597
Ventricular escape	0 (0.0)	2 (14.3)	7.517	0.006^*^
Ventricular fibrillation	0 (0.0)	2 (14.3)	7.517	0.006^*^
T wave changes	7 (13.7)	0 (0.0)	2.153	0.142
QRS low voltage	1 (2.0)	2 (14.3)	3.790	0.052
Chest X-ray, *n* (%)	*n* = 50	*n* = 15		
Enlarged heart shadow	20 (40.0)	6 (40.0)	0.000	1.000
Pulmonary edema	5 (10.0)	6 (40.0)	7.386	0.007^*^
Pleural effusion	4 (8.0)	2 (13.3)	0.392	0.531
Pneumonia	14 (28.0)	7(46.7)	1.838	0.175
Echocardiography	*n* = 48	*n* = 13		
Left-ventricular end-diastolic diameter (mm), mean ± SD	37.38 ± 5.63	37.31 ± 9.84	− 0.032	0.974
End-systolic left-ventricular posterior wall thickness (mm), mean ± SD	8.77 ± 2.69	9.54 ± 3.48	0.738	0.471
Left atrial diameter (mm), mean ± SD	20.19 ± 3.96	21.77 ± 3.06	1.546	0.135
Left-ventricular end-systolic diameter (mm), mean ± SD	25.58 ± 5.52	27.31 ± 6.59	0.865	0.399
Ventricular septum (mm), mean ± SD	6.48 ± 1.89	6.77 ± 1.48	0.588	0.562
End-diastolic left-ventricular posterior wall thickness (mm), median (IQR)	6.00 (5.00–7.00)	6.00 (5.00–8.50)	− 0.597	0.553
Left-ventricular end-diastolic volume (mL), mean ± SD	59.96 ± 22.89	64.92 ± 32.21	0.521	0.610
Left-ventricular end-systolic volume (mL), median (IQR)	23.00 (17.00–36.00)	25.00 (18.00–48.00)	0.765	0.455
Left-ventricular short axis shortening rate (%), median (IQR)	31.5 (22.25–37.00)	20.00 (13.00–37.00)	− 1.210	0.231
Left-ventricular ejection fraction (%), mean ± SD	57.29 ± 14.46	44.92 ± 19.47	− 2.534	0.014^*^
Cardiac output (L/min), median (IQR)	3.27 (2.60–4.65)	3.60 (1.14–5.33)	0.239	0.812
Pericardial effusion, *n* (%)	6 (12.5)	2 (15.4)	0.006	0.937
Congestive heart failure, *n* (%)	1 (2.1)	4 (30.8)	9.361	0.002^*^
Myocardial enzyme, median (IQR)	*n* = 48	*n* = 15		
CK-MB (IU/L)	76.00 (29.75–147.00)	379.00 (60.00–2345.00)	− 3.661	0.001^*^
cTnI (ng/mL)	3.23 (1.38–7.15)	6.26 (0.33–13.60)	− 0.501	0.623
NT-proBNP (pg/mL)	14,798.50 (5425.50–34,041.50)	14,185.00 (5790.00–35,000.00)	− 1.030	0.320
CRP (mg/L)	8.00 (8.00–16.00)	9.00 (8.00–20.00)	0.902	0.373
White blood cell count (× 10^9^/L)	9.05 (6.15–11.88)	10.00 (6.80–16.70)	− 1.791	0.088
Serum lactate (mmol/L)	2.40 (1.58–5.53)	7.40 (1.20–16.00)	− 4.591	< 0.001^*^

*ECG* electrocardiogram, *CK-MB* creatine kinase-MB, *cTnI* cardiac troponin-I, *NT-proBNP* N-terminal pro-brain natriuretic peptide, *CRP* C-reactive protein, *SD* standard deviation, *IQR* interquartile range. ^*^Statistically significant difference between two groups

Sixty-four (95.5%) children received a serological pathogen examination. Thirty-one (48.4%) children tested positive for viral infections, including nine children with coxsackie B (14.1%), five children with Epstein‒Barr virus (7.8%), four children with respiratory syncytial virus (6.3%), three children with herpes simplex virus (4.7%), three children with parvovirus (4.7%), two children with enterovirus (3.1%), two children with parainfluenza virus (3.1%), two children with adenovirus (3.1%), and one child with cytomegalovirus (1.6%). Among these 31 children, 22 (44.9%) were in the survival group, and nine were in the death group, with no statistically significant difference between the two groups (*P* > 0.05) (Table 3).

Of the 67 children, 65 (97.0%) received ECG examination. Abnormal ECG findings were more commonly seen in the death group than in the survival group, including ventricular tachycardia, ventricular escape, and ventricular fibrillation. Sixty-five (97.0%) children underwent chest X-ray examinations. The number of children with pulmonary edema in the death group was significantly higher than that in the survival group. Sixty-one (91.0%) children underwent ECG examinations. The death group had more frequent congestive heart failure than the survival group. Myocardial enzyme tests were performed in 63 (94.0%) children. CK-MB and serum lactate levels in the death group were significantly higher than those in the survival group. In contrast to the results above, ST-T segment changes were more frequently observed in the survival group than in the death group.

### Mortality prediction

**Fig. 1:**
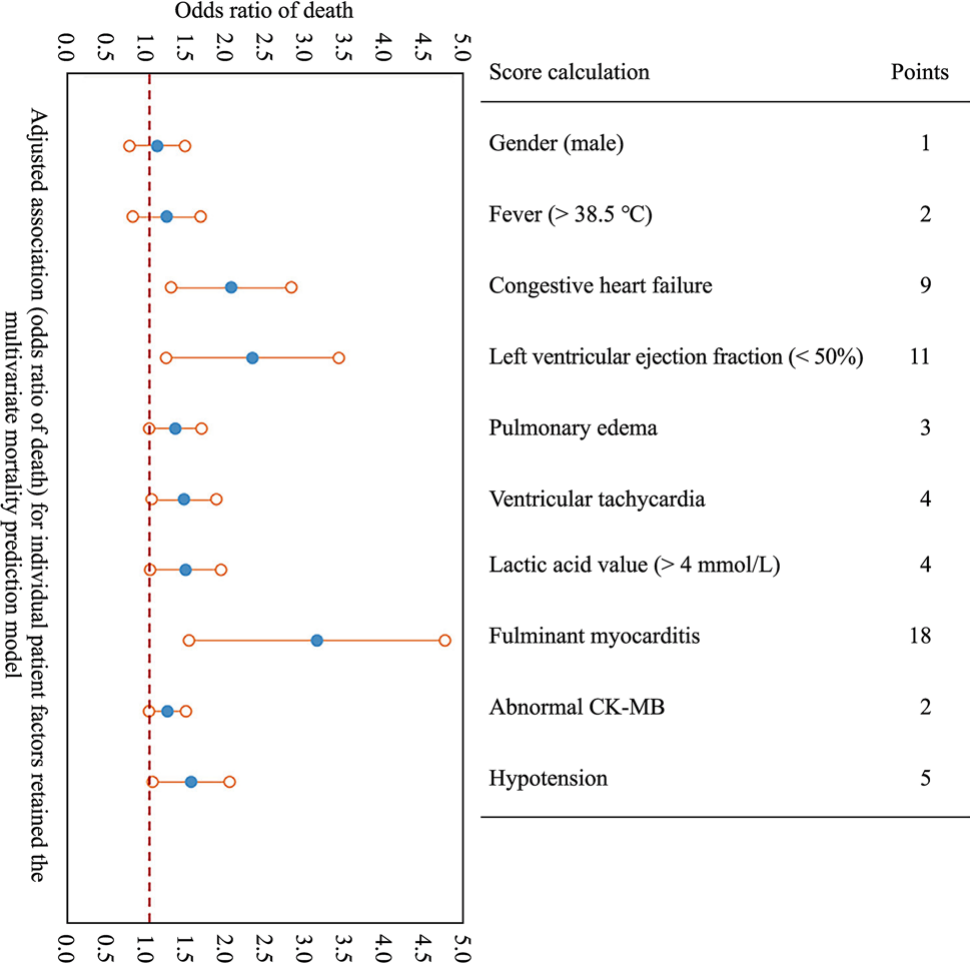
Adjusted odds of individual risk factors with death and score calculation. *CK-MB* creatine kinase-MB

**Fig. 2:**
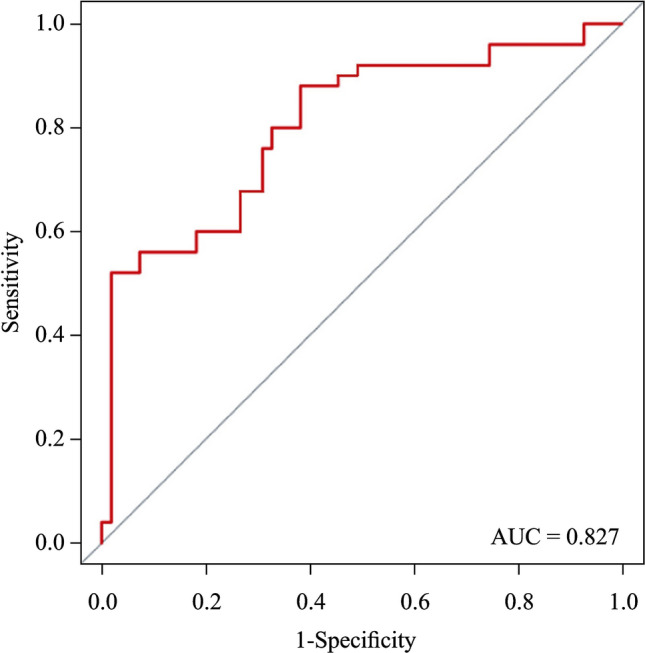
The area under the curve (AUC) of the full multivariable prediction model

**Fig. 3:**
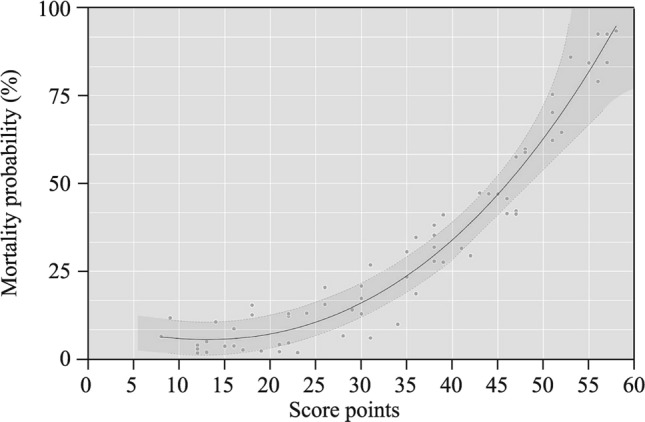
Predicted probability of death across points. Curve with 95% confidence interval shading showed the association between score points and mortality of in-hospital death among the derivation cohort

**Fig. 4:**
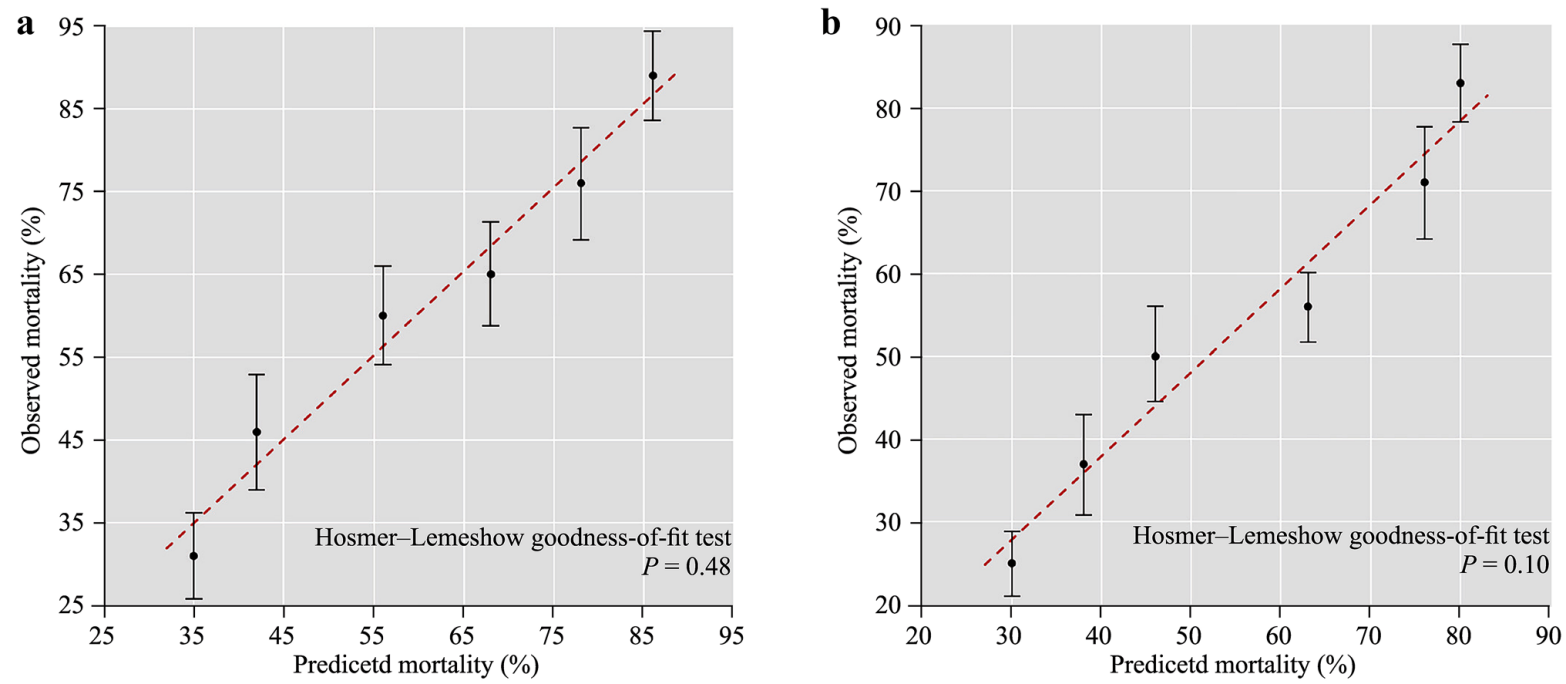
Calibration plot of observed (Y-axis) versus predicted (X-axis) mortality. **a** Correlation between observed mortality in our dataset (Y-axis) and predicted mortality according to the AMCDRS mortality prediction score (X-axis) in the derivation dataset. Discrimination and calibration of the model were among 44 patients from the Children’s Hospital of Fudan University; **b** correlation between observed mortality in our dataset (Y-axis) and predicted mortality according to the AMCDRS mortality prediction score (X-axis) in the external validation dataset. Discrimination and calibration from external validation among 44 patients with AMC from the Children’s Hospital of Fudan University. *P* > 0.05 indicates an acceptable fit. *AMCDRS* acute myocarditis death risk score, *AMC* acute myocarditis

Among factors initially associated with mortality in univariate analysis, ten factors remained in the final multivariable model (Fig. 1). The model had good discrimination, with an AUC of 0.827 (95% CI = 0.732–0.911), implying that the score could predict mortality with 83% accuracy (Fig. 2). The aforementioned variables were combined to develop a score to predict hospital survival (Table 3) and resuscitation using AMCDRS. Points are assigned according to the 10 variables, from 1 to 59. A greater number of points corresponded to a higher probability of death, which ranged from 0 to 0.99 (Fig. 3). The summed points from each of the 10 variables were used to determine the probability of death for a patient with AMC. Model calibration, to show how accurately the model fits the observed data (Hosmer–Lemeshow goodness-of-fit, *P*¼ = 0.48), indicated acceptable fit (Fig. 4a).

### Sampling validation

The predictive model was sampled and validated in 22 children with AMC from the Children’s Hospital of Fudan University who met the criteria for analysis. Children were well matched on characteristics (Table 4), including gender, fever > 38.5 ℃, congestive heart failure, left-ventricular ejection fraction < 50%, pulmonary edema, ventricular tachycardia, lactic acid value > 4, fulminant myocarditis, CK-MB abnormalities, and hypotension. Despite differences in the characteristics of the validation cohort, the model discrimination was only marginally lower, with an AUC of 0.781 (95% CI = 0.675–0.852) compared with the derivation cohort. Model calibration likewise indicated acceptable fit (Hosmer–Lemeshow goodness-of-fit. *P*¼ = 0.10) (Fig. 4b). Other bin sizes were likewise tested without improvement in fit.

**Table 4:** Comparison of derivation and validation patient characteristics

Terms	Derivation (*n* = 44)	Validation (*n* = 22)	*P*
Gender (male)	26 (65.0)	14 (69.2)	0.722
Fever (> 38.5 ℃)	17 (39.5)	9 (40.9)	0.915
Congestive heart failure	2 (4.6)	2 (9.1)	0.407
Left-ventricular ejection fraction (< 50%)	13 (29.5)	8 (36.4)	0.575
Pulmonary edema	7 (15.9)	4 (18.2)	0.535
Ventricular tachycardia	9 (20.5)	3 (13.6)	0.377
Lactic acid value (> 4 mmol/L)	25 (56.8)	14 (63.6)	0.595
Fulminant myocarditis	28 (63.6)	10 (45.5)	0.159
Abnormal CK-MB	33 (75.0)	17 (77.3)	0.839
Hypotension	11 (25.0)	7 (31.8)	0.558

Values are presented as *n* (%). *CK-MB* creatine kinase-MB

## Discussion

AMC, also known as inflammatory cardiomyopathy, is a focal or diffuse myocardial inflammatory disease associated with myocardial cell degeneration and necrosis [15]. AMC is not only a common critical inflammatory disease in the pediatric population but also a common cause of heart failure in children [16]. Viral infection is the most common cause of AMC. In this study, 64 (95.5%) children were admitted to the hospital and received serological examination. Among them, 31 (48.4%) tested positive for viral infections.

Clinical presentation in children with AMC varies significantly. No obvious symptoms may present in children with mild disease, but arrhythmia, heart failure, and even death can occur in those with severe illness. The clinical presentation of AMC varies among different age groups. Because the symptoms of AMC are nonspecific, it is difficult to make a timely diagnosis, which can result in a missed diagnosis rate as high as 83% at the first visit to the hospital [16–18]. In this study, digestive system symptoms were the most common presentation, but there were no significant differences in digestive, respiratory, and circulatory symptoms between the survival group and death group. Among 33 children with cardiogenic shock in this study, most were in the death group, which was significantly higher than that in the survival group, indicating a poor prognosis in children with AMC and severe hemodynamic instability.

Fulminant myocarditis often occurs in critically ill children with AMC and is characterized by a sudden onset of severe hemodynamic instability following viral infection. There were 38 children with fulminant myocarditis in this study, including 23 and 15 children in the survival and death groups, respectively. Furthermore, an analysis of mortality showed that 68.8% of children in the death group died within 7 days after admission, indicating that fulminant myocarditis progresses rapidly. Therefore, early clinical diagnosis and timely intervention are very important to save the lives of children with fulminant myocarditis.

Abnormal results from ECG, echocardiography, and myocardial zymogram tests are important diagnostic criteria for AMC. In this study, 65 children underwent ECG, with abnormal results identified in 64 of them. There were significantly more children with ventricular tachycardia, ventricular escape, and ventricular fibrillation in the death group than in the survival group, indicating that children with AMC who have abnormal ECG results at the time of hospital admission could have a poor prognosis. Echocardiography plays an important role in assessing cardiac function in children with and ruling out other causes of heart failure. Systolic dysfunction is common in children with AMC, characterized by a shortening fraction in the left ventricle and a lower ejection fraction, with wall motion abnormalities [12, 19, 20]. The left-ventricular ejection fraction in the survival group was significantly higher than that in the death group, indicating that cardiac function at the time of hospital admission was closely related to the prognosis. Abnormal myocardial enzyme levels are an important index for diagnosing fulminant myocarditis and evaluating the severity of AMC. In this study, CK-MB and serum lactate levels in the death group were significantly higher than those in the survival group, suggesting that these markers could be closely related to the poor prognosis in children with AMC.

Among the factors initially associated with mortality in univariate analysis, 10 factors remained in the final multivariable model, which included male sex, fever > 38.5℃, congestive heart failure, left-ventricular ejection fraction < 50%, pulmonary edema, ventricular tachycardia, lactic acid value > 4, fulminant myocarditis, abnormal CK-MB, and hypotension. The model had good discrimination, with an AUC of 0.827 (95% CI = 0.732–0.911), implying that the score could predict mortality with 83% accuracy. The predictive model was sampled and validated in 22 children with AMC from the Children’s Hospital of Fudan University who met the criteria for analysis. Children were well matched on characteristics. Despite differences in the characteristics of the validation cohort, the model discrimination was only marginally lower, with an AUC of 0.781 (95% CI = 0.675–0.852) compared with the derivation cohort. Model calibration likewise indicated acceptable fit. Several previous studies have reported different models to predict mortality in children with myocarditis. Chou et al. [22] used a large database with 2695 pediatric myocarditis cases to create prediction models for mortality. This model had an AUC of 0.934, with a low sensitivity of 55.3% and a high specificity of 95.9%. They then created another model based on the machine learning algorithm. The second model only included five variables [mechanical ventilation, cardiac arrest, extracorporeal membrane oxygenation (ECMO), acute kidney injury, and ventricular fibrillation] and had balanced sensitivity (89.9%) and specificity (85.8%). Othman et al. [14] performed a multivariate logistic regression analysis and found that several variables, such as ventricular arrhythmia, ECMO, mechanical ventilation, heart transplant, and ventricular assist device, were associated with in-hospital mortality in children with AMC. However, they did not calculate the receiver-operator characteristic curve. Kim et al. [2] reported a multivariate regression analysis, which included left-ventricular end-diastolic size, ECMO, and epinephrine infusion, to predict mortality in children with AMC. Compared with these previous models and regression analyses, our model did not include any treatments applied to the children with AMC. This was because we collected the data at the timepoint when these children were admitted to the hospital. They had not yet received advanced treatments, such as ECMO or medication infusion. The advantage of our model was that it might provide a better understanding of mortality prediction at the early stage of the disease when a physician initially evaluates a child. If considering the different treatments received after hospital admission, the mortality prediction model could certainly change. This requires physicians to closely monitor critically ill children with AMC and consider the influences of different variables during different stages of the disease process.

The limitations of our study included its retrospective design, single-center design, and small number of patients. We made the diagnosis of AMC based on widely accepted diagnostic criteria used in China [13]. There is no consensus on the diagnosis of pediatric AMC worldwide. In general, magnetic resonance imaging (MRI) and endomyocardial biopsy (EMB) are considered the most accurate methods. However, both MRI and EMB are not available or feasible in critically ill children in clinical practice. In addition, during resuscitation of children with AMC, any movements or anesthesia for additional procedures, such as MRI or EMB, might cause adverse events or exacerbate clinical conditions. We only had access to short-term in-hospital data without follow-up information after hospital discharge. In addition, we did not collect data on the management of these children, because our objective was to provide a model to facilitate mortality prediction and decision-making at the time of hospital admission. Thus, the children in this study had not yet received any advanced management of AMC. We acknowledge that any in-hospital management of AMC could certainly alter the clinical course and survival of these children. Future prospective studies are required to validate and modify our study model during different stages of the disease process.

In conclusion, we found that multiple factors were associated with increased mortality in children with AMC. AMCDRS, which includes tachycardia, fulminant myocarditis, cardiogenic shock, hypotension, and pulmonary edema, might be used at the time of hospital admission to accurately identify children with AMC who are at an increased risk of death. Further studies to validate and modify the clinical application of this model are warranted.

## Data Availability

The datasets generated and analyzed during the current study are available from the corresponding author upon reasonable request.
